# Experimental and Numerical Study on the Mechanical Behavior of Composite Steel Structure under Explosion Load

**DOI:** 10.3390/ma14020246

**Published:** 2021-01-06

**Authors:** Kai Zheng, Xiangzhao Xu

**Affiliations:** State Key Laboratory of Explosion Science and Technology, Beijing Institute of Technology, Beijing 100081, China; 154306216@bit.edu.cn

**Keywords:** composite steel structure, mechanical behavior, explosion load, numerical simulation

## Abstract

Most engineering structures are composed of basic components such as plates, shells, and beams, and their dynamic characteristics under explosion load determine the impact resistance of the structure. In this paper, a three-dimensional composite steel structure was designed using a beam, plate, and other basic elements to study its mechanical behavior under explosion load. Subsequently, experiments on the composite steel structure under explosion load were carried out to study its mechanical behavior, and the failure mode and deformation data of the composite steel structure were obtained, which provided important experimental data regarding the dynamic response and mechanical behavior of the composite steel structure under explosion load. Then, we independently developed a parallel program with the coupled calculation method to solve the numerical simulation of the dynamic response and failure process of the composite steel structure under explosion load. This program adopts the Euler method as a whole, and Lagrange particles are used for materials that need to be accurately tracked. The numerical calculation results are in good agreement with the experimental data, indicating that the developed parallel program can effectively deal with the large deformation problems of multi-medium materials and the numerical simulation of the complex engineering structure failures subjected to the strong impact load.

## 1. Introduction

Designing high-value targets such as those for large buildings, bridges, and protection projects is necessary, as it ensures their safety under a strong impact load. Thus, the study of the dynamic response of structures under explosion load has important research value [1,2,3]. Most engineering structures are composed of basic components such as plates, shells, and beams, and their dynamic characteristics under explosion load will determine the impact resistance of the structure [4,5,6,7,8]. Therefore, studying the dynamic response of these basic components under explosion load has important engineering application value for predicting the dynamic response of structures under strong impact load and improving the anti-explosion ability of structures [9,10,11,12].

Extensive research on beams, slabs, columns, and other structures regarding the explosion and impact damage of beam–slab composite structures has been conducted. Ning et al. [4] provide a novel theory to predict the transient deformation behavior of the thin shell under impact load using the energy method and the variational principle; they found that the deformation region has the pit shape characteristics, and they provide the variation law of the pit radius and depth with the projectile velocity. This theory is efficient and concise, and it has important significance for the study of the mechanical behavior of the thin shell under impact load. Nemirovskij and Romanova [13] studied the failure morphology analysis of sheet metal components subjected to explosion and impact loads under viscoelastic boundary conditions. Woznica et al. [14] conducted an explosion experiment indoors and obtained the corresponding failure data of metal plate components. Curry and Langdon [15] studied the explosion effect when several metal plates were stacked together. Park et al. [16] carried out an explosion resistance experiment on a steel plate and obtained its strain and deflection data from a 50 kg TNT explosion. Remennikov et al. [8] performed a detonation test using a short-range nitromethane spherical explosion on a steel plate, and they obtained the pressure and displacement of the steel plate. Zheng et al. [17] investigated the dynamic response of unreinforced steel and reinforced steel plate under limited explosion load tests and measured the deformation profile of the sample. Yao et al. [18] conducted an internal explosion experiment on a steel box and obtained the dynamic response process of the steel box girder under the internal explosion load and the propagation process of internal shock waves. Zhang et al. [19] carried out internal explosion tests on steel box models with different loading conditions, different structural sizes or different structural materials, and observed two failure modes: convex damage and concave damage.

Numerical simulation is not limited by experimental scenarios, and it can realize simulation analysis of problems under any scenarios to obtain the changing law of different parameters. The data can be visualized through post-processing methods; thus, we can obtain a clearer and more intuitive understanding of the propagation process of the explosion shock wave in the explosion problem, and we can also gain a deeper understanding of the internal mechanism, which has important guiding significance for experimental research [4,12,20,21,22]. The explosion damage experiment of the composite steel structure in this work is relatively difficult, and the experiment requires more funds and time, thus numerical simulation is particularly important.

For the numerical simulation of 3D multi-medium interaction subjected to explosion load, Ning et al. [2] innovatively proposed a 3D fuzzy interface method, in which the multi-medium interface is not determined, and the volume ratio of the material calculated is considered as a fuzzy weight coefficient by which transport quantities are determined. Compared with other interface treatment methods, the fuzzy interface treatment can conveniently deal with three or more mixed grids in the three-dimensional area, and the calculation cost is lower. The fuzzy method changes the mode of traditional interface treatment methods. It is a representative method in the Euler method to track the material interface and provides an efficient numerical simulation method for the dynamic response mechanism of materials and structures under explosion and impact load. In addition, Feng [23] established a dynamic finite difference calculation model for simulating large deflection and conducted dynamic response analysis of hinged steel beams under flame and explosion loads. Song et al. [24] investigated the dynamic response of X70 steel pipes under local explosion load and performed the numerical simulations based on experimental data to analyze four different failure modes. Thanh et al. [21] studied the deformation behavior of a square steel plate under explosion load through the LS DYNA numerical method using three different kinds of modeling methods. Micallef et al. [22] used ABAQUS/Explicit to simulate and analyze the response of a circular thin steel plate under local explosion load at a close range and identified the permanent lateral displacement for different scenarios. Qi et al. [25] established a new type of steel constitutive model considering fire (high-temperature softening effect) and explosion (strain rate effect), and they conducted numerical simulation analysis for the collapse process and mechanism of steel frame structures under the combined action of high temperature and explosion. Mehreganian et al. [26] conducted a finite element model combined with experiments to analyze the response of low carbon steel and armored steel under a limited charge explosion, and they obtained the transient deformation of the plate, the localization of strain, and the pressure distribution of the plate. Ma et al. [27] studied the dynamic fracture failure of AISI 1045 steel pipe under internal explosion load and analyzed the expansion and fracture process of the cylinder using LS-DYNA. Currently, although certain research results have been obtained for the numerical solution of the dynamic response of the composite structure under the explosion load, a single algorithm cannot effectively deal with this. Current research work requires additional algorithms, which reduces the accuracy and computational efficiency. For example, Ma et al. [28] proposed a coupled VOF (volume of fluid) and PIC (particle in cell) algorithm that is suitable for the two-dimensional multi-material interface of the Euler method, which can track the material deformation process in the penetration problem. Liu et al. [29] proposed the MGFM (modified ghost fluid method) to construct the Riemann problem at the interface and correct the state of the virtual fluid. This method can perform high-precision numerical simulations of underwater explosions and the interaction between shock waves and structures.

In this paper, a three-dimensional composite steel structure is designed using a beam, plate and other basic elements to study its mechanical behavior under explosion load. Subsequently, a mechanical behavior experiment on the composite steel structure under two types of explosion loads with and without explosion height is carried out. This experiment uses high-speed photography to record its action process, and it obtains the failure mode and deformation data of the composite steel structure. The effects of explosion height on the result of the explosion are analyzed. This test provides important experimental data for the dynamic response and damage of the steel structure under explosion load. An independently parallel program is developed to conduct the numerical simulation of the mechanical behavior of the composite steel structure under explosion load. This program is based on the Euler method, adding Lagrange particles to the Euler grids for materials that need to be tracked accurately, and it uses the bidirectional mapping to realize the mapping of physical quantities between the Euler grid and the Lagrange particles. The numerical calculation results are in good agreement with the experimental data, indicating that the developed parallel program can effectively deal with the large deformation problems of multi-medium materials and the numerical simulation of the complex engineering structure failures subjected to the strong impact load.

## 2. Experiments

High-speed photography technology was used to obtain the dynamic failure process of the composite steel structure and the explosion shock wave propagation process. The damage to the composite steel structure after the explosion was analyzed, and the damage degree of each part of the composite steel structure was obtained.

### 2.1. Design of the Composite Steel Structure

**Figure 1:**
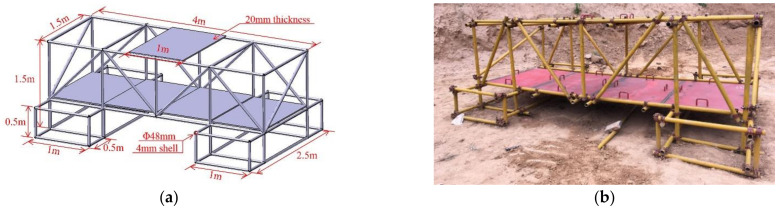
Composite steel structure. (**a**) Three-dimensional size; (**b**) Site construction.

The composite steel structure is composed of scaffold steel pipes and steel plates. Regarding the dimensions of the scaffold steel pipes, their diameter is 48 mm, and their wall thickness is 4 mm. The steel plate material is 1045 steel, and it is 1.5 m in length, 1.0 m wide in width, and 20 mm in thickness. The steel plates weigh 234.0 kg, and each steel plate is processed with four handles to provide convenience for the movement and position adjustment during erection. A counterweight steel plate is placed on the middle of the upper steel pipe of the composite steel structure, and the material is the same as the steel plate. A specific size design and construction diagram are shown in Figure 1.

### 2.2. Explosive Design

**Figure 2:**
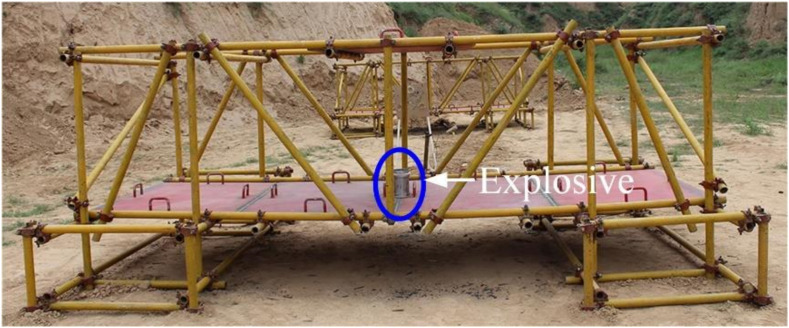
Position of the explosive for the scenario without explosion height.

**Figure 3:**
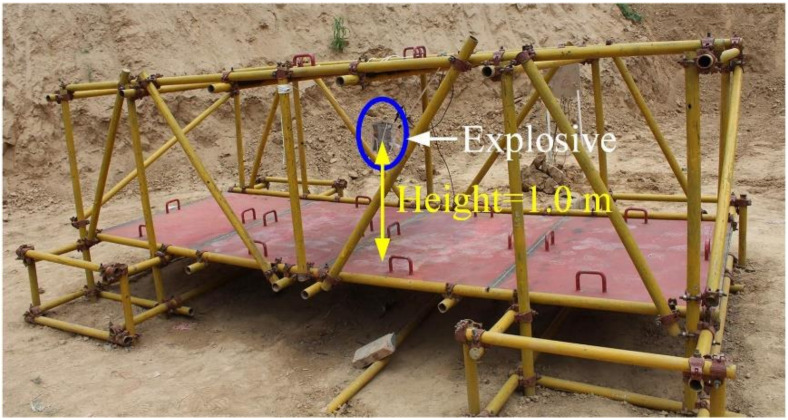
Position of the explosive for the scenario with a certain explosion height.

The explosive used in the experiment was a shelled cylindrical explosive B. The inner diameter of the cylindrical explosive shell was 15.0 cm, the outer diameter was 16.0 cm, the wall thickness was 0.5 cm, the inner charge height was 17.4 cm, and the total height was 19.4 cm. The charge of explosive was 3.50 kg. In the experiment, the same composite steel structure was used to set up two scenarios with different explosive positions. Scenario 1: the explosive is placed at the center of the steel plate deck, as shown in the blue circle note in Figure 2; scenario 2: the explosive is placed 1.0 m higher than in scenario 1, as shown in the blue circle in Figure 3. These two scenarios are described as the experiment without explosion height (scenario 1) and the experiment with a certain explosion height (scenario 2).

### 2.3. Experimental Results

#### 2.3.1. Experiment without Explosion Height

**Figure 4:**
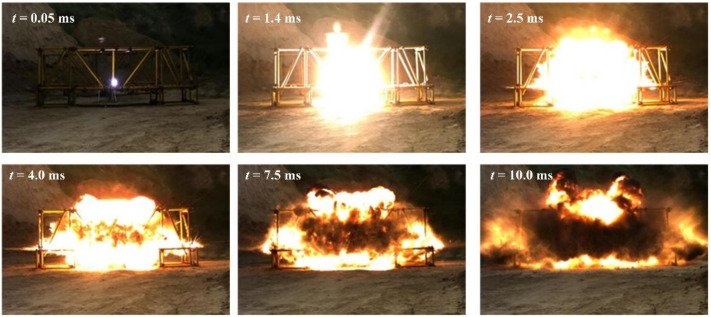
The explosion process without explosion height.

Based on the design scenario in Figure 2, the scenario of the explosion experiment without explosion height was built, and the explosion process was photographed by high-speed photography, as shown in Figure 4. At the beginning of the explosion, the detonation product had extremely high energy and rapidly expanded from the explosive in a high-bright state; then, the energy of the detonation product decreased, and the propagation speed rapidly decreased. The detonation product changes from a high-brightness state to a black smoke-like state, and the explosion shock wave propagated to the surroundings at a faster speed.

**Figure 5:**
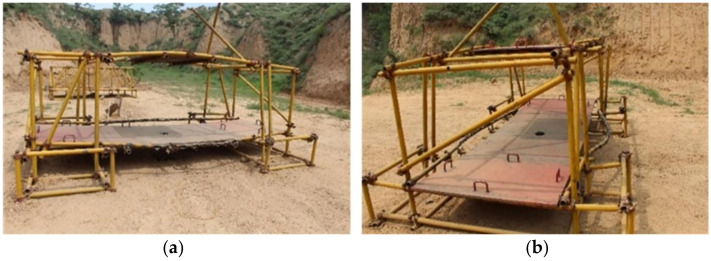
Damage to composite steel structure after the explosion without explosion height. (**a**) Front view; (**b**) Side view.

Figure 5 shows the damage to the composite steel structure after the explosion without explosion height. The closer the structure is to the explosive, the greater the degree of bending. The curvature of the center of the composite steel structure is greater than that at the end. A circular hole with the same size as the explosive appeared where the explosive was placed, indicating that shear plugging occurred in the steel plate during explosive detonation. The tubes on both sides of the composite steel structure were severely bent outward or even fractured. The three sets of steel pipes at the middle positions on both sides of the composite steel structure lost their support and protection. The steel pipes in other positions of the composite steel structure that were also bent lost some of their functionality of support and protection. The closer the structure is to the explosion center, the greater the degree of bending and the worse the functionality.

The measurement results show that the most severe upward bending of the steel plate above the composite steel structure was about 13.0 cm. The most severe downward bending of the steel plate under the composite steel structure was about 12.8 cm. The steel plate shown below had a circular penetration hole with a diameter of about 15.5 cm, which is approximately circular. There were 16 handles on the bridge before the explosion, while, following it, there were only 8. In total, 50% of the handles were knocked off. This was mainly due to the stress caused by the explosion shock wave propagating in the steel plate.

#### 2.3.2. Experiment with a Certain Explosion Height

**Figure 6:**
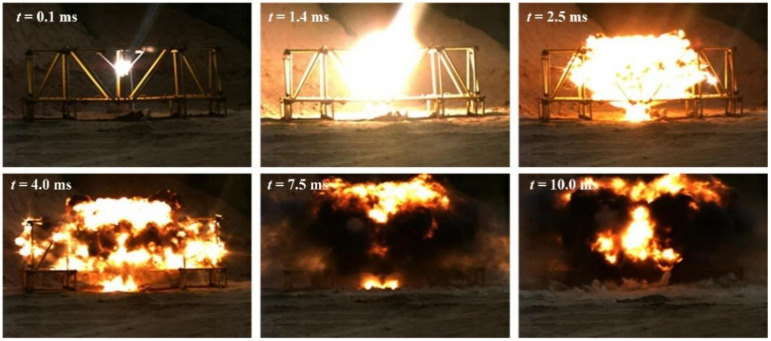
The explosion process with a certain explosion height.

Based on the design scenario in Figure 3, the scenario of the explosion experiment with a certain explosion height at a distance of 50.0 cm from the surface of the steel plate was built, and the explosion process was photographed by high-speed photography as shown in Figure 6. The high-speed photography results show that the explosion process is similar to the experiment without explosion height.

**Figure 7:**
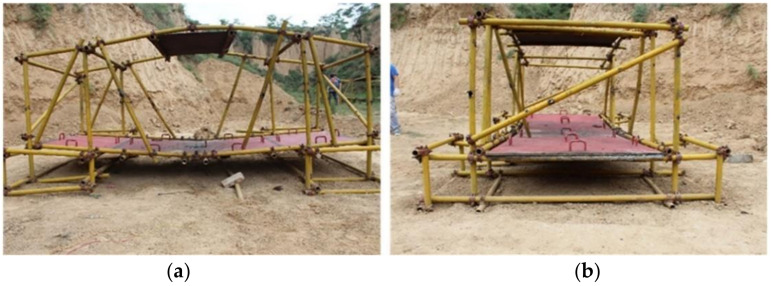
Damage to the composite steel structure after the explosion with a certain explosion height. (**a**) Front view; (**b**) Side view.

Figure 7 shows the damage to the composite steel structure after explosive detonation with a certain explosion height. The measurement results show that the most severe bending of the steel plate above the composite steel structure was about 17.0 cm, and the steel plate below the composite steel structure was bent by about 14.2 cm. The steel plate of the composite steel structure after explosive detonation was more severe in the case of explosion height than in the case of no explosion height. The difference in the degree of bending of the upper steel plate was more prominent. It is preliminarily speculated that the explosive shock wave damage effect produced by the explosive detonation of the scenario with explosion height is better than that of the scenario with no explosion height. In the scenario with a certain explosion height, only one handle of the steel plate shown below was knocked off, but the bending degree of the plate was higher than that in the scenario without explosion height. It is speculated that the handle was knocked off due to the shock wave inside the steel plate. As for the overall impact of the explosion shock wave on the steel plate, the scenario with a certain explosion height was more intense than that without it.

Based on the above analysis, the following phenomena occurred during the damage process of the two scenarios: at the beginning of the explosion, the explosive product expanded rapidly from the explosive in a high-bright state; then, the propagation speed gradually decreased, and the explosive product changed from the high-bright state to black smoke. The explosion shock wave propagated to the surrounding area at a faster speed. Both the upper and lower steel plates of the composite steel structure were obviously bent. The steel pipe structures that make up the composite steel structure displayed bending and fractures. For the damage to the upper and lower steel plates of the composite steel structure, the damage effect of the scenario with a certain explosion height was better than that of a scenario without it. For the damage to the steel pipe structure of the composite steel structure, the damage effect of the scenario without explosion height was better than that of a scenario with a certain explosion height.

## 3. Numerical Method

The mechanical behavior of the composite steel structure under explosion load involves large deformation and failure of material. The EXPLOSION-3D software was developed independently by our research group [2,28,30,31]. This software is a three-dimensional multi-material Eulerian engineering simulation software using the finite difference method. It is a parallel software capable of large-scale computation and can reach the computational scale of hundreds of millions of grids on an eight-node cluster, with each node possessing two Intel E5620 CPUs; the memory is 32 GB, and every CPU has six cores in it. It has significant advantages in dealing with the large deformation of materials, the interface of various materials and various strong discontinuous explosion and impact problems, and it can effectively simulate the complex explosion and impact process in engineering. Moreover, while this software was developed based on the Euler method, can solve the large deformation problem well, and has obvious advantages in dealing with the explosion damage of the composite steel structure, it encounters difficulties in accurately tracking the failure and deformation of materials. To solve these problems, a coupled method with the Lagrange method is introduced in this paper, which effectively combines the advantages of the Euler method and the Lagrange method to realize accurate tracking of material deformation history, and, on this basis, further numerical simulation research on the failure behavior of the composite steel structure under explosion load is carried out.

### 3.1. Governing Equation

In order to better describe the physical process of the dynamic response of the composite steel structure under explosion load, the non-conservative Euler equations can be expressed as follows when the external force, external source, and heat conduction are ignored [30]:

(1) $$\frac{\partial \rho }{\partial t}+u\cdot \nabla \rho +\rho \nabla \cdot u=0$$

(2) $$\frac{\partial u}{\partial t}+u\cdot \nabla u=\frac{1}{\rho }\nabla \cdot \sigma$$

(3) $$\rho (\frac{\partial e}{\partial t}+u\cdot \nabla e)=\sigma \cdot \dot{\varepsilon }$$

where t is time, u is velocity, σ is Cauchy stress tensor, ρ is density, and e is specific internal energy.

The stress tensor can be composed of the deviatoric stress and the hydrostatic pressure tensor, whose expressions are as follows:

(4) σ=−PI+S

where P is the hydrostatic pressure calculated by the state equation, I is the unit tensor, and S is the deviatoric stress tensor obtained by the constitutive equation.

### 3.2. Constitutive Equation

The fluid elastic-plastic model is adopted, the material stress tensor is composed of hydrostatic pressure and deviatoric stress, and the strain is composed of elastic strain and plastic strain. The specific expression is as follows [30]:

(5) $$\sigma_{ij}=-PI_{ij}+S_{ij}$$

(6) $$e_{ij}=e_{ij}^{e}+e_{ij}^{p}=(\frac{e_{ii}}{3}\delta_{ij}+\varepsilon_{ij}^{e})+\varepsilon_{ij}^{p}$$

In Equation (5), $PI_{ij}$ represents the volume change of the material, and the volume change caused by this is the elastic volume change, which does not lead to shape change of the material. $S_{ij}$ represents the actual deformation degree of the material and follows the incremental elastic-plastic stress–strain relation. The rate of change is calculated by the stress rate.

### 3.3. Equation of State

In this paper, the numerical simulation of the dynamic response of the composite steel structure under explosion load involves metal, air and explosive materials. In the numerical simulation, the following equations of state are used to describe each material:

(1) Metal materials

The Mie–Grϋneisen state equation [32] is used to characterize the dynamic mechanical behavior of metal materials under impact loading and its expression is as follows:

(7) $$P=P_{H}(1-\frac{\Gamma \mu }{2})+\Gamma \rho \left(e-e_{0}\right)$$

in which

(8) $$P_{H}=\left\{\begin{array}{ll} k_{1}\mu +k_{2}\mu^{2}+k_{3}\mu^{3}, & \mu \geq 0\\ k_{1}\mu , & \mu <0 \end{array}\right.$$

where Γ is the Grϋneisen coefficient, $k_{1}$, $k_{2}$, and $k_{3}$ are the constants related to material performance; $\mu =\rho /\rho_{0}-1$, $\rho_{0}$ is the initial density of the metal before impact; ρ is the real-time density; $e_{0}$ is the unit mass specific internal energy of the metal before impact; e is the real-time specific internal energy. The metal material involved in this paper is 1045 steel, and its equation of state parameters are shown in Table 1.

(2) Air material

In numerical calculation, the state equation of ideal gas [33] is used to calculate the air material, and its expression is as follows:

(9) $$P=\;\left(k_{a}-1\right)\rho \cdot e$$

where ρ is the air density, e is the specific internal energy of the air, $k_{a}$ is the isentropic index of the air, and $k_{a}=1.4$ is used in numerical calculation.

(3) Explosive

The JWL state equation [33] is adopted for detonation products generated after explosive detonation. The expression is as follows:

(10) $$P=A(1-\frac{\omega }{R_{1}V})e^{-R_{1}V}+B(1-\frac{\omega }{R_{2}V})e^{-R_{2}V}+\frac{\omega e}{V}$$

where V is the volume ratio of the detonation product and unexploded explosive, and A, B, $R_{1}$, $R_{2}$ and ω are undetermined constants. Explosive B is used in this paper, and the calculation parameters of its equation of state are shown in Table 2.

### 3.4. Yield and Failure Criteria

Numerical simulation of the dynamic response of the composite steel structure under explosion load involves large deformation of materials. Therefore, it is necessary to modify the plastic state of the material. The deviatoric stress correction factor β is defined. If the plastic yield deformation of the material occurs, then 0<β<1, and the specific value of the material needs to be corrected according to the test value of deviator stress. If the material occurs when elastic deformation, then β=1.

(1) Yield criterion of metal materials

The yield of metal material was described using Von-Mises yield criteria. The yield conditions are as follows:

(11) $$J_{2}=\frac{1}{2}S_{ij}S_{ij}=\frac{1}{3}Y_{0}^{2}$$

If $J_{2}<\frac{1}{3}Y_{0}^{2}$, the material is in the elastic stage; thus, the stress–strain relationship can be obtained by the generalized Hooke’ s law. If $J_{2}\geq \frac{1}{3}Y_{0}^{2}$, the material enters the plastic stage; thus, the deviatoric stress correction factor can be calculated by the following formula:

(12) $$\beta =\frac{Y_{0}}{\sqrt{\frac{3}{2}S_{ij}S_{ij}}}$$

The yield strength $Y_{0}$ can be modified according to the thermal softening effect; that is, the yield strength $Y_{0}$ decreases with the increase in specific internal energy e. The specific yield strength correction formula is as follows:

(13) $$Y_{0}=\left\{\begin{array}{ll} Y_{0}(1-e/e_{m}), & e<e_{m}\\ 0, & e\geq e_{m} \end{array}\right.$$

where $e_{m}$ is the melting specific internal energy.

(2) Criterion of instantaneous tensile failure

The failure thresholds $P_{s}$ and $\rho_{s}$ for hydrostatic pressure and material density are defined. When the grid pressure or material density exceeds the failure threshold, the materials in the grid will fail instantly. Meanwhile, both hydrostatic pressure and deviator stress in the grid are set to 0, namely:

(14) $$P_{ijk}=\left\{\begin{array}{cccc} P_{ijk} & (P_{ijk}>P_{s} & \mathrm{and} & \rho_{ijkl}>\rho_{s})\\ 0 & {(}_{s}P_{ijk}\leq P & \mathrm{or} & \rho_{ijkl}\leq \rho_{s}) \end{array}\right.$$

(15) $$S_{ijk}=\left\{\begin{array}{cccc} S_{ijk} & (P_{ijk}>P_{s} & \mathrm{and} & \rho_{ijkl}>\rho_{s})\\ 0 & (P_{ijk}\leq P_{s} & \mathrm{or} & \rho_{ijkl}\leq \rho_{s}) \end{array}\right.$$

### 3.5. Operator Splitting Algorithm

In this paper, the operator splitting algorithm is adopted [2]. According to the physical characteristics of the three governing equations, they can be divided into an influence of diffusion phase and a convection phase. Therefore, the three governing equations can be uniformly represented as:

(16) $$\frac{\partial \varphi }{\partial t}+u\cdot \nabla \varphi =H$$

where φ represents physical quantities, u·∇φ is the convection phase, and H is the diffusion phase.

According to physical effects, Equation (16) can be divided into two stages: the Lagrangian stage and the Eulerian stage. In the Lagrangian stage, the effect of the pressure gradient and the deviator stress is considered, while the influence of the convection phase is ignored, causing the mesh changes with material deformation, pressure, and velocity. In the Eulerian stage, physical quantities of mass, momentum, and energy are reallocated by calculating transport volumes between grids.

### 3.6. Material Interface Treatment Method for Accurate Tracking

The Euler method is the preferred method to solve the problem of large deformation of materials subjected to strong impact load, and its core idea is to use a fixed space coordinate system and the material through the mesh boundary. The numerical simulation of large deformation does not directly influence the material. Thus, it is able to deal with the large deformation problem, but the interface between the multi-material is difficult to track, and the accuracy of tracking the material interface is not as good as that of Lagrange methods [33]. In this paper, a coupled Euler and Lagrange method, which combined the advantages of the Euler method and Lagrange methods, is adopted [34]. In this method, the particle with a certain influence domain is used to track the material inside the Euler grid, and the bidirectional mapping is used to realize the mapping of physical quantities between the Euler grid and the Lagrange particles. With Equation (17), the physical quantity $Q_{L}$ of the Lagrange particles can be obtained from the physical quantity $Q_{E}$ of the Euler grid, where Q can represent the physical quantities such as mass, momentum, volume, and energy:

(17) $$Q_{L}=\sum_{n=1}^{8}\frac{V_{\alpha }}{\sum_{l=1}^{N}V_{l}}Q_{E}$$

where N is the total number of all Lagrange particles in the influencing domain in the current grid. $V_{\alpha }$ is the volume of the particle influence domain in the surrounding grids. $V_{l}$ is the volume occupied by the particle in the current grid.

After the mapping of physical quantities from the Euler grid to the Lagrange particles, the velocity of the Lagrange particles can be obtained with the following volumetric weighting formula:

(18) $$U_{L}=\sum_{n=1}^{8}\frac{V_{n}}{\sum_{l=1}^{N}V_{l}}U_{E}$$

where $U_{L}$ and $U_{E}$ are the velocities of the Lagrange particles and Euler grid, respectively.

Then, the position coordinates of the Lagrange particles at the next moment can be obtained. After the above steps, the Lagrange particles move to a new position, changing the original position relationship with the Euler grid. With the sum of the Lagrange particles $N^{\prime }$ in the current grid, the physical quantity $Q_{E}^{\prime }$ of the Euler grid after the Lagrange particles move can be obtained:

(19) $$Q_{E}^{\prime }=\sum_{l=1}^{N^{\prime }}\frac{V_{\alpha }^{\prime }}{V_{l}}Q_{L}$$

where $V_{\alpha }^{\prime }$ is the volume of the influence domain in the surrounding grids after the particles move.

After the above steps, the transport of physical quantities between Euler grids is realized through the movement of the Lagrange particles. This method overcomes the numerical fluctuation caused by the finite number of particles and provides it with improved computational performance. Moreover, there is no interpenetration between different materials due to the fixed grid and single mapping. The coupled Euler and Lagrange method effectively avoids the defects of particles in the cell methods, maintains its original advantages, and improved the accuracy of tracking material interface.

### 3.7. Material Interface Treatment Method without Accurate Tracking

For material interface treatment without accurate tracking, this paper adopts the fuzzy interface method [2] to deal with the transport of physical quantities between grids. The volume proportion of each kind of material in the grid volume is calculated, and it is used as the fuzzy weight coefficient to determine the transport volume. Meanwhile, according to the priority of material transport, the transport sequence of various materials is determined. Compared with other interface treatment methods, the fuzzy interface treatment can deal with three or more mixed grids in the three-dimensional area conveniently, and the calculation cost is lower.

In the fuzzy interface method, the transport priority of each material is determined based on the fuzzy comprehensive evaluation method [2]. Assuming λ represents a physical quantity of material existing in the current grid, the physical quantity of the material in the adjacent left and right grids is $\eta_{L}$ and $\eta_{R}$, respectively. If λ does not exist, $\eta_{L}$ or $\eta_{R}$ are assigned to 0; if λ exists, $\eta_{L}$ or $\eta_{R}$ are assigned to 1. The volume ratio of the current grid to the left and right grids are defined as $V_{\lambda L}$ and $V_{\lambda R}$, respectively. The values of $0\leq V_{\lambda L}\leq 1$ and $0\leq V_{\lambda R}\leq 1$ can be obtained according to the definition. Based on $\eta_{L}$, $\eta_{R}$, $V_{\lambda L}$, and $V_{\lambda R}$, the physical quantities of the current grid transport to the left $Q_{\lambda L}$ and right grids $Q_{\lambda R}$ can be calculated:

(20) $$Q_{\lambda L}=\eta_{L}\cdot \mathrm{sgn}(V_{\lambda R}-V_{\lambda L})$$

(21) $$Q_{\lambda R}=\eta_{R}\cdot \mathrm{sgn}(V_{\lambda R}-V_{\lambda L})$$

Considering all possible combinations of $Q_{\lambda L}$ and $Q_{\lambda R}$, the distribution of materials can be divided into five cases, as shown in Table 3. Once the distribution of a material is obtained, its transmission priority can be determined according to the continuity principle. The transmission priority is $\lambda_{1}>\lambda_{2}>\lambda_{3}>\lambda_{4}>\lambda_{5}$. Subsequently, the physical quantity needed to be transported is equal to the product of the transport factor κ and the original physical quantity. The calculation formula of κ is as follows:

(22) $$\kappa =\left\{\begin{array}{ll} 0.5\left(V_{id}+V_{ic}\right), & V_{ic}\neq 0\\ V_{ic}, & V_{ic}=0\\ 1, & V_{ic}=1 \end{array}\right.$$

where $V_{id}$ and $V_{ic}$ are the volume ratios of material η in the contribution grid and the receiving grid, respectively.

The finite difference interpolation method is used to implement the numerical method in this paper, and MPI (message passing interface) standard design is adopted to carry out modular programming for the parallel programs. The three-dimensional parallel program can realize large-scale parallel computation of complex problems, and its computational scale can reach the level of hundreds of millions of grids. Algorithm 1 summarizes the procedure for the calculation of the module works.
**Algorithm 1 Flow Chat of the Calculation Module Works.**1: Input the computational data of MESH-3D pre-processing software; 2: Model data initialization and parallel initialization; 3: **if** (the materials in the grid are those that require accurate tracking) **then**; 4: Particle physical quantity initialized; 5: **end if** 6: **while** (the calculation time is less than the set time); 7: Computational domain boundary processing module: it is mainly used to calculate the physical quantities of the boundary grids and particles in the computational domain; 8: Lagrangian stage calculation module; 9: **if** (material interface treatment method for accurate tracking) **then**; 10: Coupled Euler and Lagrange method calculation module (Section 3.6); 11: **else** (material interface treatment method without accurate tracking) 12: Fuzzy interface method calculation module (Section 3.7); 13: **end if** 14: Equation of state calculation module (Section 3.3); 15: **end while** 16: Output calculation result module

## 4. Numerical Analysis

### 4.1. Numerical Calculation Model

**Figure 8:**
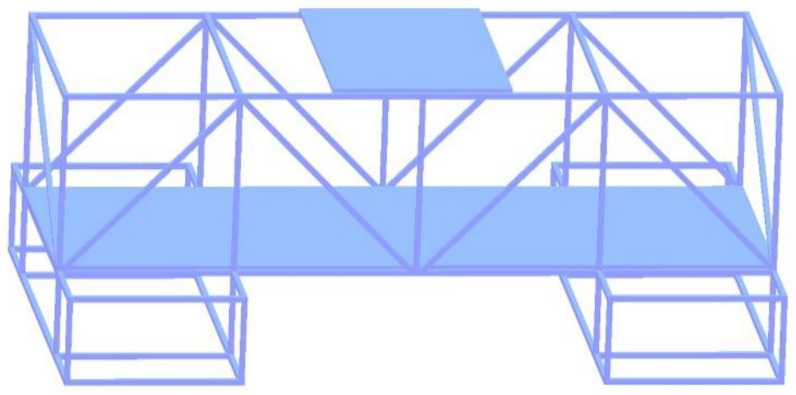
Numerical calculation model of composite steel structure.

The numerical calculation model of the composite steel structure is shown in Figure 8. The charge of explosive B is 3.5 kg with a density of 1.717 g/cm^3^ and a detonation velocity of 7980 m/s. The size of the composite steel structure is consistent with the experiment.

**Figure 9:**
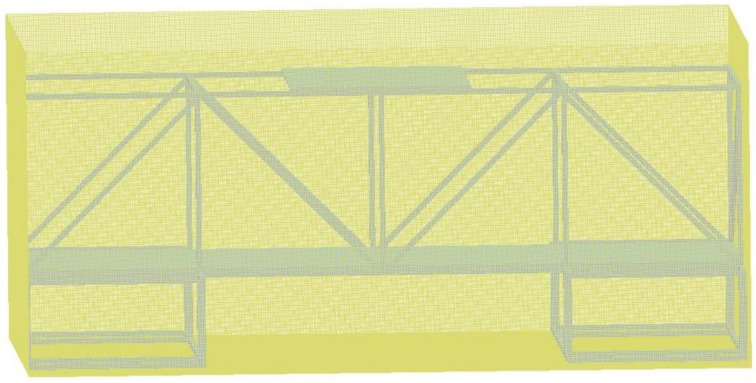
Mesh model.

The numerical calculation of a three-dimensional explosion field often involves complex structures, and it is difficult to meet the practical engineering needs with manual modeling. To solve the problem of complex structure modeling, a MESH-3D pre-processing software [35,36] was developed by our research group according to the characteristics of the calculation program. This software can be arbitrarily complex structure for rapid grid generation and display, and it can provide good pretreatment data for the numerical calculation. Thus, the pre-processing uses MESH-3D software [35,36] to mesh the composite steel structure model, and the results are shown in Figure 9. The numerical calculation method in this paper is based on the finite difference method. In order to facilitate the calculation, the model adopts a regular hexahedral mesh of equal step length. The length of each grid in both directions is 0.8 cm, and the total number of grids is 57.65 million. After importing the calculation model in the pre-processing stage, the following information is determined: (1) The size of the calculation domain and the grid step size are set. (2) The boundary of the calculation domain is a continuous boundary. (3) Three kinds of materials are set. (4) Select 1045 steel material is selected, the equation of state is the Mie–Grϋneisen equation of state, and the corresponding parameters are input as shown in Table 1. The explosive material is explosive B, the equation of state is JWL, and the corresponding parameters were input as shown in Table 2. Air is an ideal gas, and the equation of state is an ideal gas. A four-node cluster server is used for numerical calculation. Each node has two Intel E5620 CPUs (each CPU has six cores) and 32 GB memory, and the calculation explosion process is set to 10.0 ms.

### 4.2. Numerical Results

**Figure 10:**
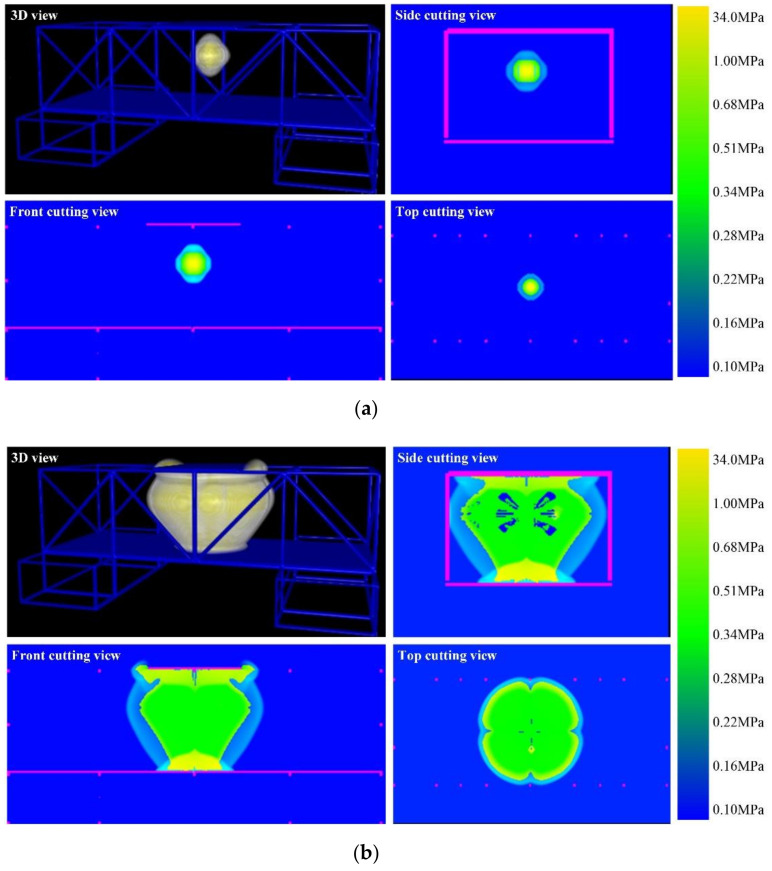

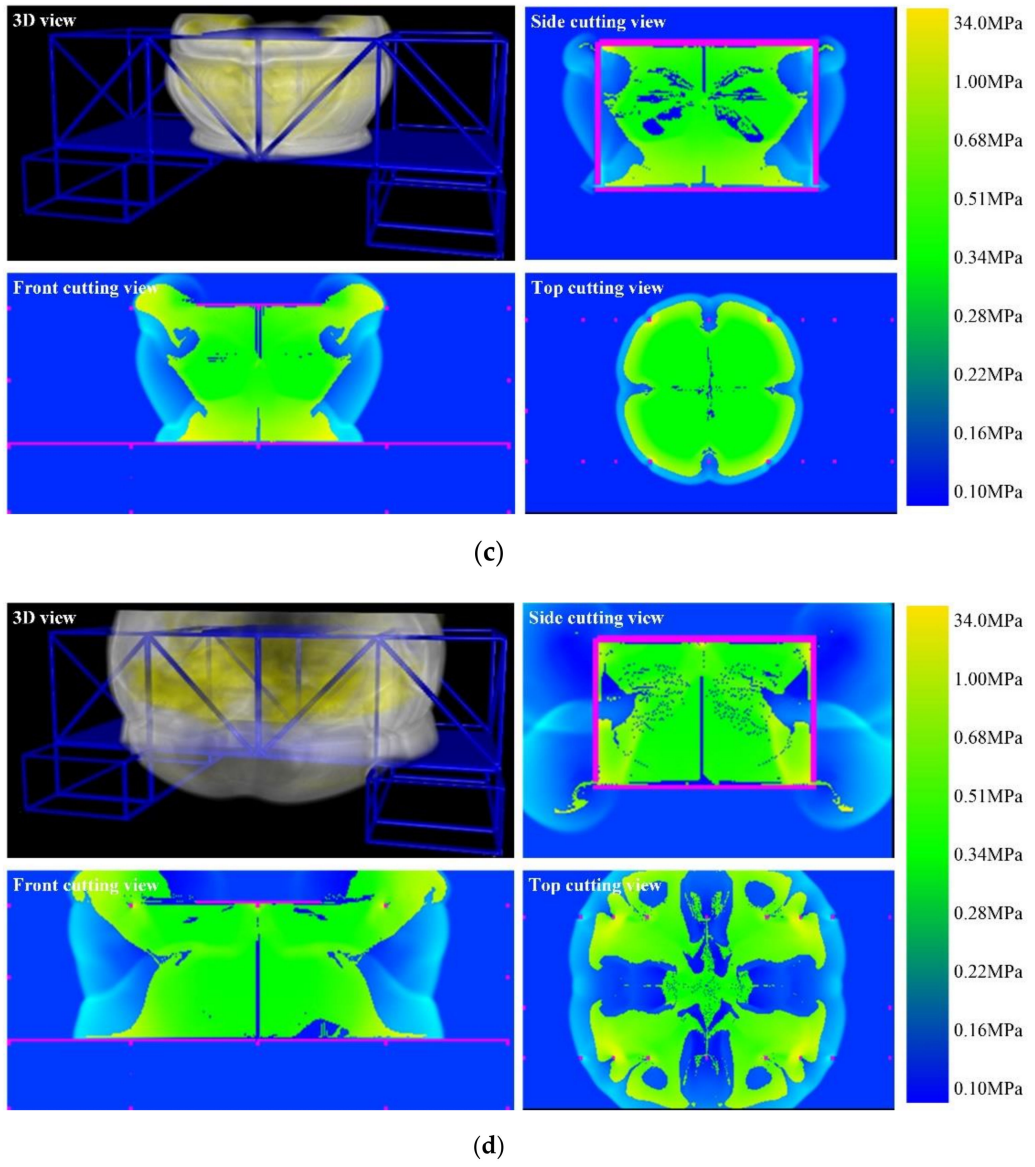
Explosion shock wave propagation images at different times with a certain explosion height. (**a**) *t* = 0.20 ms; (**b**) *t* = 1.80 ms; (**c**) *t* = 4.0 ms; (**d**) *t* = 8.0 ms.

**Figure A1:**
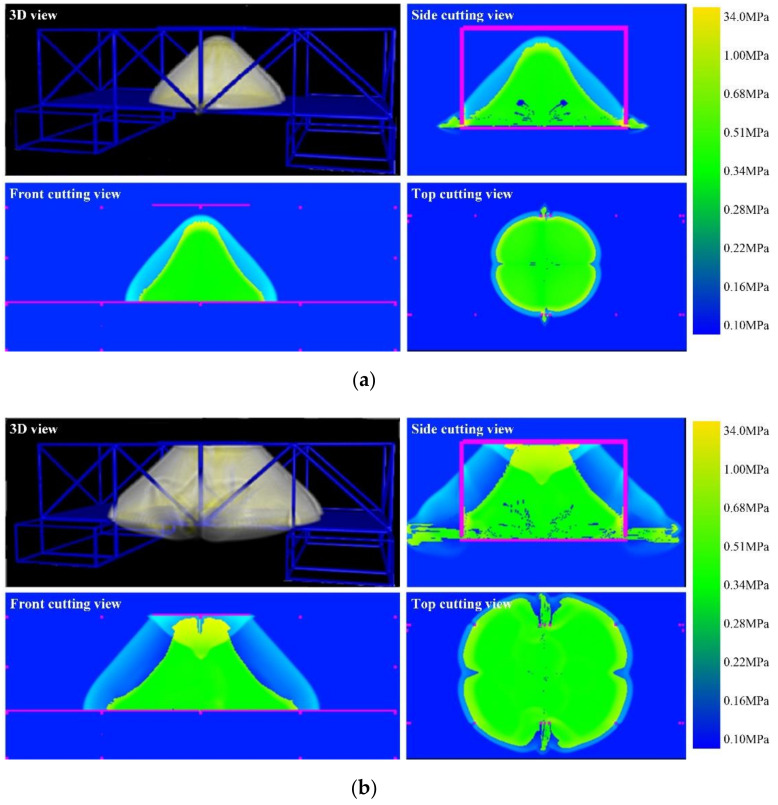

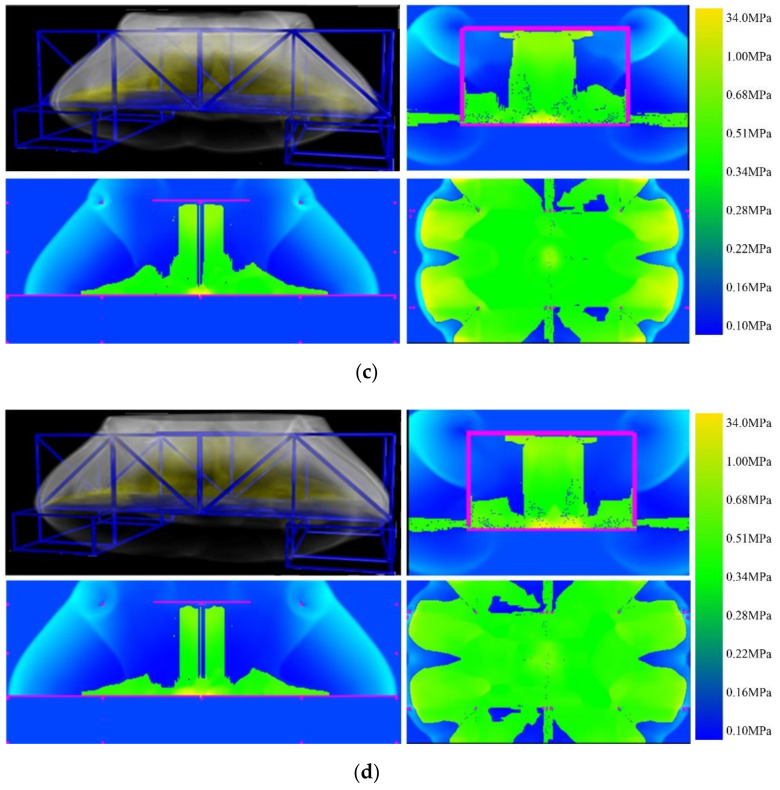
Explosion shock wave propagation images at different times without explosion height. (**a**) *t* = 1.80 ms; (**b**) *t* = 4.00 ms; (**c**) *t* = 8.00 ms; (**d**) *t* = 10.00 ms.

Figure 10 shows the propagation process of the explosion shock wave at different times. After explosive detonation, the high pressure and temperature gas explosion products rapidly expand outward, impacting and compressing nearby air, causing the pressure and density of the compressed air to increase sharply. Due to the reflection and diffraction of the steel tube and steel plate, the composite steel structure is impacted by multiple shock waves. According to Figure 10, the explosion shock wave propagates extremely fast in the first 2.0 ms, and then, as the pressure decreases, the velocity of the explosion shock wave slows down. The results of the scenario without explosion height are similar to those of the scenario with a certain explosion height. The numerical simulation results are shown in the attached Figure A1.

In each case of numerical simulation, gauge points are set at key positions, and the pressure value of each gauge point is monitored at each moment to reflect the pressure on each position, thereby reflecting the damage at each key position. The damage law inside the composite steel structure is analyzed by the comparative analysis of the pressure values at the gauge points of the same row or different rows. The damage results in the different scenarios are obtained by the comparative analysis of the pressure time history curves of the gauge points at the same position in the different scenarios.

**Figure 11:**
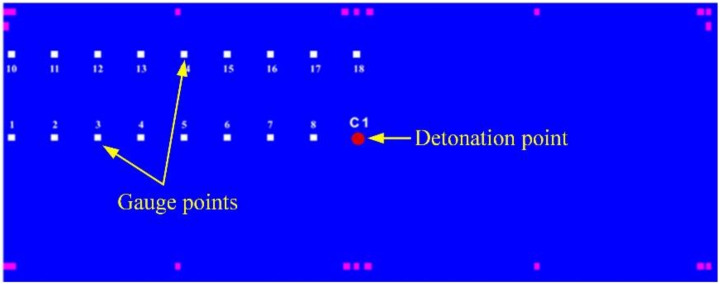
Initiation point and gauge point position.

The gauge points used to monitor the pressure value are shown in Figure 11. The peak pressure changes with the distance from the explosion center are reflected by the comparison of the peak pressures at different gauge points. The red dot C1 represents the cylinder of 3.5 kg explosive B, the numbers 1–8, and the other rows 10–18 represent the total 17 gauge points, and each gauge point distance in the same row is 25.0 cm.

**Figure 12:**
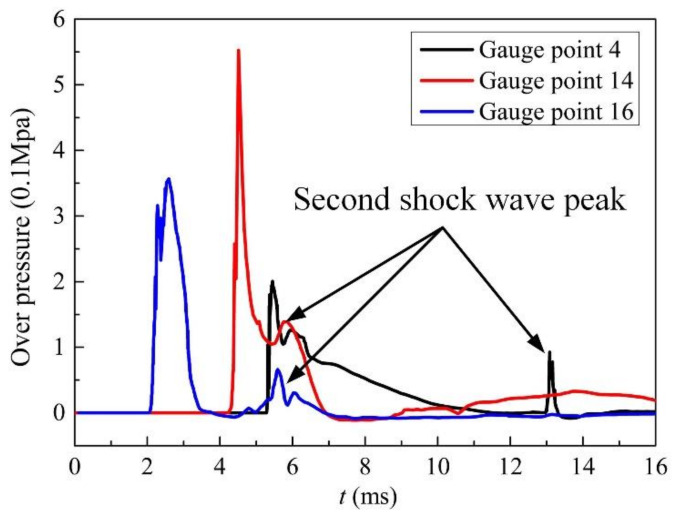
Pressure vs. time curves of some gauge points in air.

Figure 12 shows the pressure vs. time curves of some gauge points. The pressure vs. time curves of three gauge points clearly shows the moment when each point is affected by the first detonation shock wave, and the detonation shock wave makes the pressure of each point rise instantly to the maximum peak pressure. As shown in Figure 12, there is more than one peak pressure at each gauge point. The reason is that the superposition of shock waves from different directions and the reflected waves from the steel plate or tube increases the pressure at that place, but these pressure values are less than the previous peak pressure value. In the evaluation of the damage effect of explosives, the peak pressure value is an important parameter. Therefore, in the follow-up work of this article, the peak pressure value will be studied in more detail.

**Figure 13:**
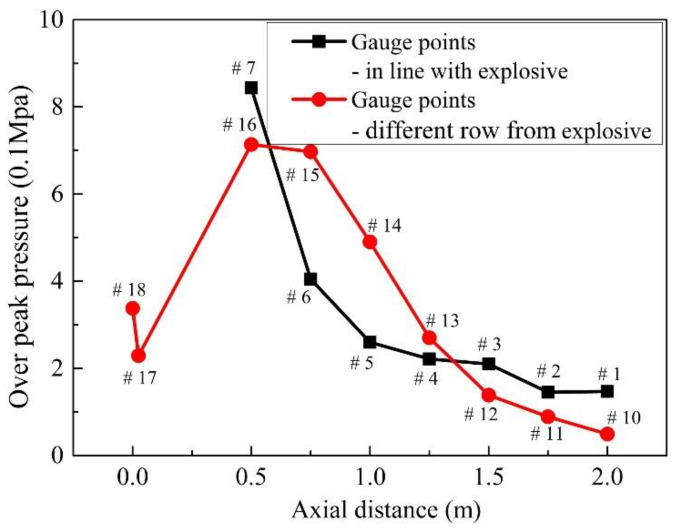
The peak pressures of gauge points in air.

A comparison of the peak pressures of gauge points in the same row is shown in Figure 13. The trend of peak pressure values at gauge points (1–8) in the same row of explosives reflects the peak pressure within 1.0 m from the explosion center decreases rapidly with the increase in the distance that between the gauge point and the explosion center. In the range of 1.0 m–2.0 m from the explosion center, the peak pressure decreases slowly, and the damage capability is significantly reduced. The change trend of the peak pressure at the other row of gauge points (10–18) shows that the pressure peaks of the gauge points 15 and 16 are relatively high due to the superposition of the detonation wave reflected on the steel plate.

**Figure 14:**
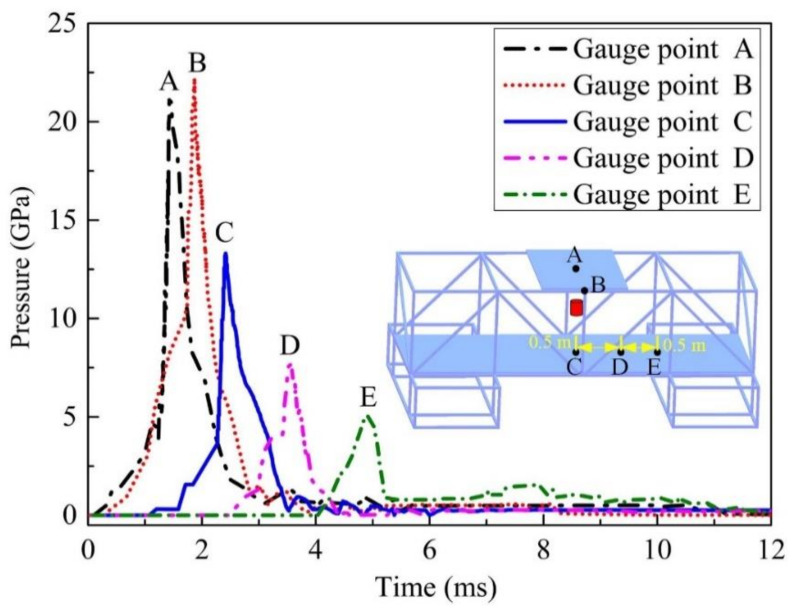
Pressure vs. time curves of some gauge points in steel.

The pressure vs. time curves of some gauge points in steel are shown in Figure 14. The pressure inside the steel plate jumps due to the high pressure produced by the explosive blasting. The peak pressure exceeds 20.0 GPa closer to the central area (Gauge A and Gauge B), and the peak pressure decreases rapidly with the increase in distance from the explosive (Gauge C, Gauge D, and Gauge E). Comparison with Figure 10 shows that the stress in the steel plate appears significantly earlier than the arrival time of the shock wave; this is because the propagation velocity of the stress wave in the steel plate is significantly higher than that of the shock wave in the air. Furthermore, in addition to a single peak pressure, there are several subsequent pressure pulsations at the gauge points, which are the result of multiple reflections of the shock wave on the steel plate within the composite steel structure. The pressure of Gauge E at a distance of 1.0 m from the horizontal direction of explosion also reached 5.0 GPa instantaneously, indicating that the elastic deformation of the steel plate under the action of explosion loading can be ignored, and it is mainly plastic deformation.

### 4.3. Comparison of Numerical Results and Experimental Data

**Figure 15:**
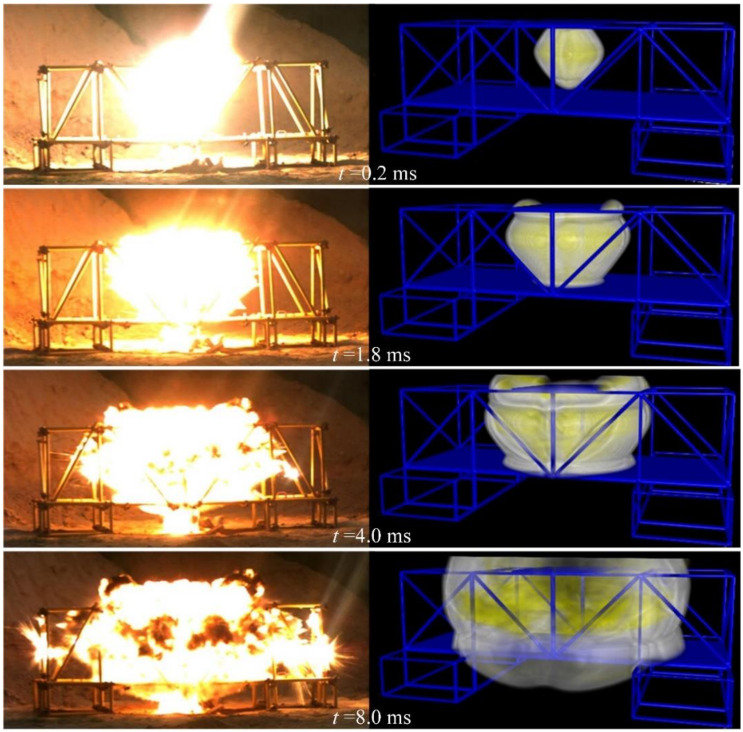
Comparison between the scenario with a certain explosion height and the experimental data.

**Figure A2:**
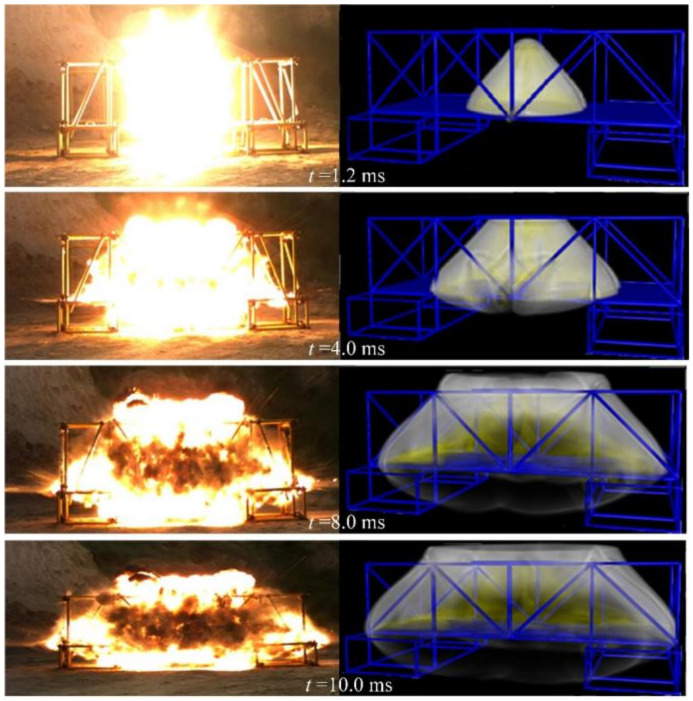
Comparison between the scenario without explosion height and the experimental data.

The propagation state of the shock wave at each moment in the simulation results is compared with the pictures taken by high-speed photography in the experiment with a certain explosion height. The results of the comparison show that the propagation law of the shock wave is consistent, as shown in Figure 15. A comparison between the scenario without explosion height and the experimental data is shown in the attached Figure A2.

**Figure 16:**
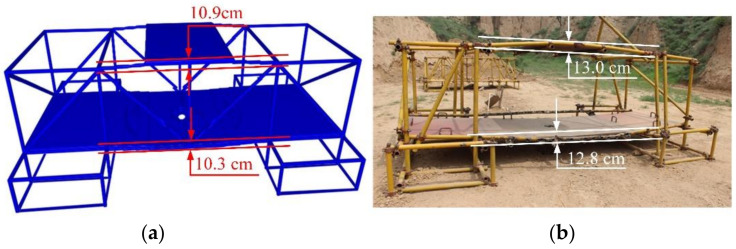
Comparison between the numerical results of the deformation of the composite steel structure and test data. (**a**) Numerical results; (**b**) Test data.

**Figure 17:**
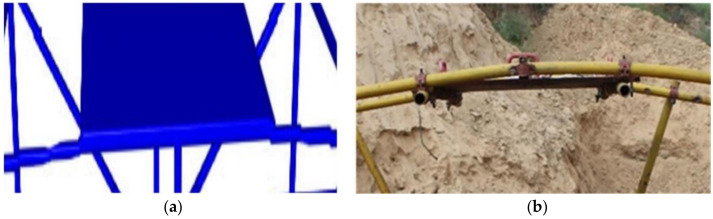
Comparison of numerical and test data of damage details. (**a**) Numerical results; (**b**) Test data.

Figure 16 shows the comparison between the numerical results of the deformation of the composite steel structure and the experimental data. Both the numerical simulation and the experiment formed a hole with the same diameter as the explosives on the steel slab where the explosive was located, and the steel slab showed significant downward bending, as shown in Figure 17. In the numerical simulation results, the steel plate on top of the composite steel structure can also be clearly observed to bend in an upward direction, which is generally consistent with the experimental results. The most severe upward bending of the steel tube was about 13.0 cm, and that obtained by numerical simulation was 10.9 cm. The most severe downward bending of the steel tube was about 12.8 cm, and that obtained by numerical simulation was 10.3 cm. The steel plate shown below had a circular penetration hole with a diameter of about 15.5 cm, and that obtained by numerical simulation was 15.2 cm. The maximum error between numerical results and experimental data was 2.5 cm, which indicates that the numerical method proposed in this paper can effectively predict the mechanical behavior of the composite steel structure under explosion load.

**Figure 18:**
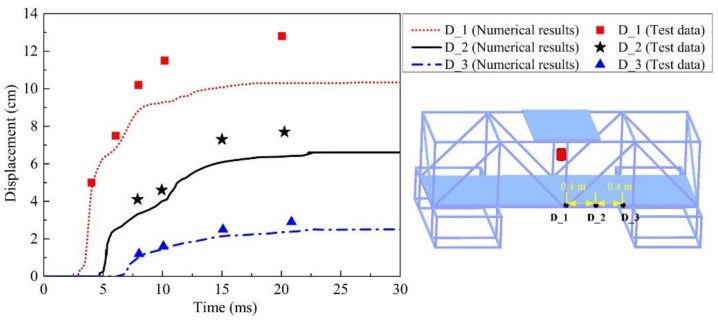
Comparison of numerical and test data of gauge points in tube.

High-speed photography can be used to measure the deformation process of a composite steel structure at different positions and at different times. Explosive detonation produces intense light, which results in the capture effect of high-speed photography. Thus, it is difficult to measure the deformation process of beam and plate structures in the nearby area. Using the results of high-speed photography, the data images were collated and analyzed to obtain the deformation history of the tube on the steel plate edge as shown below, and the deformation displacements of three-gauge points at different times were measured, as shown in Figure 18. The displacement–time curves of the three-gauge points at the same position in the numerical simulation results were extracted to compare with the test data, as shown in Figure 18. The comparison results show that the numerical simulation results are consistent with the test data within 10.0 ms. However, with the increase in time, the difference between the numerical simulation results and the test data becomes increasingly larger, and the closer the structure is to the position of the explosives, the more significant the difference. Furthermore, the deformation time of the tube is clearly dependent on the pressure action time, indicating that the deformation process of the tube is much slower than the propagation of stress.

### 4.4. Analysis of the Explosion Shock Wave Propagate in the Composite Steel Structure

#### 4.4.1. Effect of Different Explosive Positions

**Figure 19:**
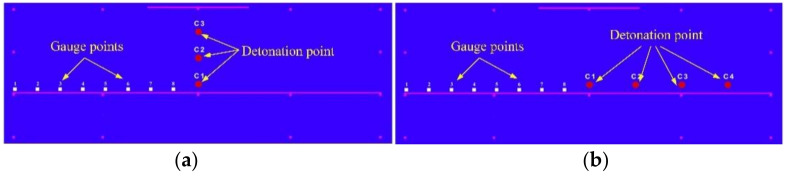
The arrangement of different explosive positions and gauge points. (**a**) Different height of the explosive position; (**b**) Different horizontal position of the explosives.

Seven scenarios with different explosive positions were set up for comparative analysis. The first comparison examines three different scenarios, and the only different parameters of the three scenarios are the height of the explosive position, the explosive position, and the gauge point positions, as shown in Figure 19. The only variables in the difference between the four cases of the second comparison are the horizontal position of the explosives, the explosive position, and the gauge point positions, as shown in Figure 19.

**Figure 20:**
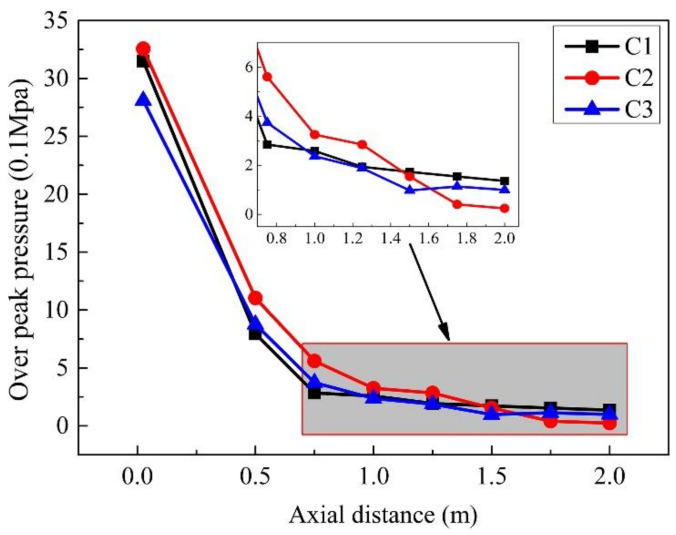
The peak pressure of the gauge points in the case of different explosion heights.

Figure 20 shows the peak pressure of each gauge point in the case of different explosion heights. For the explosive scenarios of different explosion heights, the pressure change trend at each gauge point in the three cases is the same. Comparison results show that, within the horizontal radius of 1.50 m, the damage of explosives with a certain height of explosion is more serious than that of those without a certain height or those that are too high. In the range of 1.50 to 2.00 m, the damage ability of explosives without explosion height is slightly higher. Therefore, based on the above analysis, the scenario with a certain explosion height provides a better damage effect.

**Figure 21:**
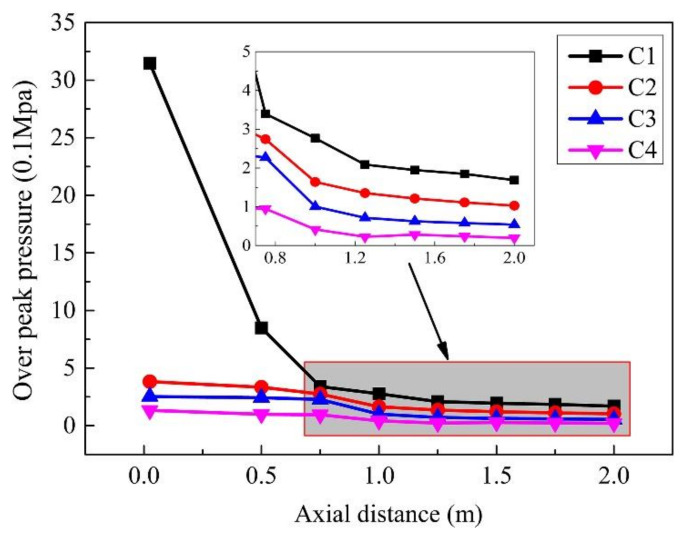
The peak pressure of the gauge points in different horizontal positions of explosives.

The peak pressure of the gauge points in different horizontal positions of explosives are shown in Figure 21. The distance shown in the figure is the distance between the observation point and the position of the C1 explosive. For different explosive horizontal positions, the attenuation trend is the same in all cases: the farther away from the explosive position, the lower the pressure. Compared with other scenarios, placing explosives at the center of the composite steel structure has the best damage effect.

#### 4.4.2. Effect of Different Explosive Weights

**Figure 22:**
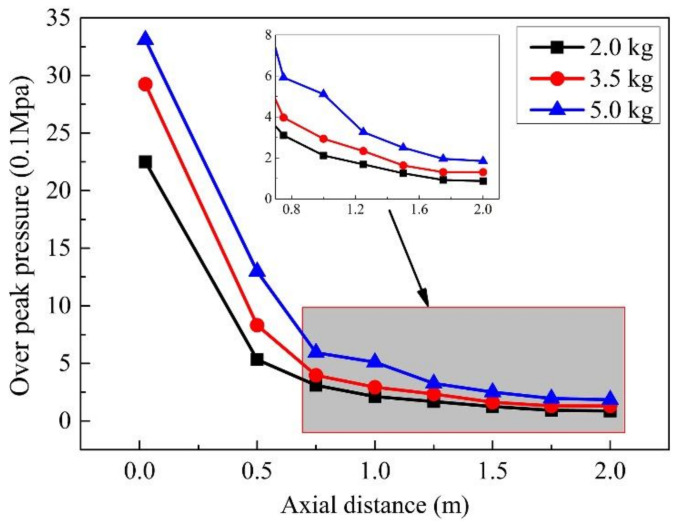
The peak pressure of each gauge point in the case of different explosive weights.

In this section, the only variable parameter of the three scenarios is explosive weight. The explosive weights of the three scenarios are 2.0 kg, 3.5 kg, and 5.0 kg, respectively. The explosion center position is the same in the three scenarios, and the gauge point position remains unchanged. The peak pressure changes at gauge points 1–8 are shown in Figure 22. The changing trend of the peak pressure at the gauge points at different positions of the three scenarios is the same. As the explosive weight increases from small to large, the peak pressure at the same position also increases. The gauge point with the lowest pressure is the #1 gauge point with an explosive weight of 2.0 kg. When the explosive weight is 2.0 kg, the peak pressure at the #1 gauge point on the steel plate farthest from the explosive center can reach 0.08 Mpa. This pressure will instantly rupture the human eardrum, damage the internal organs, and cause serious damage to the cars.

### 4.5. Damage Assessment

The action of the explosion shock wave is instantaneous. In this scenario, it takes only 10 ms for the beginning of the explosion shock wave propagation caused by explosive detonation to reach the entire composite steel structure. Therefore, if an explosion occurs, there is an immediate effect, i.e., there is no time to react and seek a proper way to avoid it. When the shock wave overpressure reaches 0.035 MPa, it causes the human eardrum to rupture and internal organs to be slightly damaged. When the shock wave overpressure is between 0.21 and 0.28 MPa, the internal organs of humans are obviously damaged. When the shock wave overpressure is between 0.70 and 0.84 MPa, the human body is unable to withstand the pressure, leading to instant death.

For the situation described in this experiment, the damage ability is dependent on the distance between the subject and explosive center. With a 1.0 m distance from the explosion center, the peak pressure decreases rapidly with increase in the distance between the reference point and the explosion center, and the pressure decreases more significantly with a 0.5 m distance from the explosion center. At a distance of 1.0–2.0 m from the explosion center, the peak pressure decreases slowly and the damage capability is significantly reduced. When the explosive weight is 3.5 kg, overpressure within a radius of 0.25 m can directly kill humans. Within a radius of 1.0 m, the minimum pressure can cause serious damage to human internal organs. For the same 3.5 kg explosive, the pressure within a 3 m radius of the explosion center will instantly rupture the human eardrum and slightly damage the internal organs, even at different horizontal positions. In addition to distance, the damage ability of the explosive is also dependent on the height. Within a radius of 1.25 m, the damage effect of a 3.5 kg explosive at an explosion height of 0.5 m is better than that at explosion heights of 0.0 m and 1.0 m. For a 3.5 kg explosive detonation at a height of 0.5 m, human internal organs within a radius of 1.0 m from the explosion center can be seriously damaged. When compared with explosion at a height of zero, the damaging range is expanded by more than 30.0%. The influence of 2.0 kg explosives is high enough to cause instantaneous rupture of eardrums and internal organ damage to humans in all areas of the composite steel structure. The 5.0 kg explosive is high enough to cause irreversible serious damage to the internal organs of humans near the composite steel structure. Furthermore, near the explosive location, the pressure on both sides of the bridge may increase due to the superposition of shock waves, while the superposition effect of the shock waves at a distance is significantly reduced.

## 5. Conclusions

Designing high-value targets, such as those for large buildings, bridges, and protection projects, is necessary, as it ensures their safety under strong impact load. Thus, the study of the dynamic response of structures under explosion load has important research value. In this paper, the dynamic response of a composite steel structure under the action of explosion load is studied, and a damage behavior experiment and numerical simulation study of the composite steel structure under explosion load are carried out. The following conclusions are obtained:

(1) The damage to the composite steel structure under explosion load is concentrated in the near field, and that in the far field is relatively small;

(2) From the perspective of damage, maximum damage is observed when the explosive is located in the center of the composite steel structure and, when it has a certain height of explosion, from the perspective of protection, the explosion shock wave will rapidly attenuate with the increase in distance. When the explosion occurs, quick evacuation is recommended.

(3) The calculation program developed in this paper can effectively simulate the dynamic response of the composite steel structure under the action of explosion load, and it can obtain the propagation law of the explosion shock wave in the complex structure and the damage law of the structure. It provides a feasible and reliable method for the subsequent numerical simulation study of the complex structure, and provides a reference for the damage and protection of the composite steel structure.

In this article, all numerical simulations are performed under ideal conditions and only consider the relevant effects of the explosion shock wave, but the actual explosion is an extremely complicated process. In order to gain a deeper understanding of the relevant information, the following aspects should be considered in future research:

(1) Actual modeling and numerical simulation of explosives, considering the damage effect of fragments on the composite steel structure and the coupling effect of shock waves and fragments;

(2) Numerical simulation of the real structure and actual material and actual size modeling to make the numerical simulation more realistic.

## Figures and Tables

**Table 1:** Mie–Grϋneisen state equation parameters.

Γ	C (cm/μs)	θ	$k_{1}$	$k_{2}$	$k_{3}$
1.69	0.3574	1.920	1.002	2.846	4.388

**Table 2:** Properties and parameters of explosive B.

ρ (g/cm^3^)	$P_{CJ}$ (GPa)	$D_{CJ}$ (m/s)	*A* (GPa)	*B* (GPa)	$r_{1}$	$r_{2}$	w
1.717	29.5	7980	542	7.68	4.2	1.1	0.24

**Table 3:** Distribution of materials.

	$\lambda_{1}$	$\lambda_{2}$	$\lambda_{3}$	$\lambda_{4}$	$\lambda_{5}$
$\eta_{L}$	0	1	−1	−1	0
$\eta_{R}$	1	1	−1	0	0
